# Factors Influencing Length of Stay in Cholecystectomy Patients in a Community Hospital

**DOI:** 10.51894/001c.6346

**Published:** 2017-12-19

**Authors:** Monica Zipple, Eliza Slama, James Wilkie, Alicia Kieninger, Robert Robinson

**Affiliations:** 1 Saint Joseph Mercy Oakland Hospital, General Surgery Resident; 2 St. Agnes Hospital, Baltimore MD, Currently a General Surgery Resident; 3 University of British Columbia, Vancouver, BC, CAN, Currently a Family Medicine Resident; 4 Core Faculty, General Surgery Residency Program

**Keywords:** gallbladder disease, length of stay, cholecystectomy

## Abstract

**CONTEXT:**

Gallstone disease is a major health problem addressed by general surgeons, with approximate incidence of 10-15% in the Western world. With increasing focus in the healthcare literature on cost containment, controlling excess lengths of hospital stay (LOS) in this population is paramount. The aim of this study was to determine the factors that influence LOS in cholecystectomy patients to examine whether results would indicate a possible improvement in perioperative patient care and decrease costs at our community hospital in a suburban setting.

**METHODS:**

This is a retrospective review during a two-year period from 1/1/2013-12/31/2014 of patients admitted from the emergency department and undergoing cholecystectomy during the same admission. The study team analyst conducted univariate analysis for significant predictors of length of stay.

**RESULTS:**

The authors identified a total analytic sample of 312 subjects who met inclusion criteria. Sample patients admitted to the surgical service had a statistically significant shorter LOS than those patients who were not (3.4 days +/- 1.7 vs 5.6 days +/- 3.0; p value <0.0005). There was also a moderate positive correlation between decreased time to surgery and LOS (Pearson R-value 0.420, p value < 0.0005). Patients admitted to non-surgical services were more likely to have comorbidities like COPD, DM, arrhythmia, CAD, anticoagulation, CHF and previous abdominal surgeries. However, when placing each comorbidity into an analysis of covariance, patients admitted to surgical services still had a significantly shorter LOS (p value < 0.0005).

**CONCLUSIONS:**

Admission to a non-surgical service and increased length of time to surgical intervention were associated with prolonged LOS and potentially increased cost in cholecystectomy patients in this study sample. Though patients admitted to non-surgical services are “sicker,” they still had prolonged LOS when controlling for comorbidities. Based on these findings, the establishment of an acute care surgery service may help to address this disparity in care.

## INTRODUCTION

Gallstone disease (i.e., cholelithiasis) is a major health problem frequently addressed by general surgeons. This condition affects approximately 10-15% of adults in the Western world population during their lives.^1,2^ Approximately 6.3 million men and 14.2 million women in the US are afflicted with the condition.^1,2^ The primary risk factors include being female, older, an ethnic/family history, obesity, metabolic syndrome, rapid weight loss, certain diseases (e.g., cirrhosis, Crohn’s disease), and gallbladder stasis.^2^ Those individuals with cholelithiasis may either be asymptomatic or experience symptoms from intermittent cystic duct obstruction (i.e., epigastric or right upper quadrant abdominal pain) after eating fatty foods, with nausea and vomiting. Evaluation in an emergency department typically includes a laboratory workup (i.e., electrolytes, renal function tests, alkaline phosphatase, liver enzyme evaluation, complete blood count) with assessment for obstructive jaundice and an abdominal ultrasound or CT scan.

Gallstone disease can present in a wide variety of clinical scenarios. Patients may experience symptomatic cholelithiasis (also known as gallbladder colic) with intermittent obstruction of the cystic duct resulting in the classic symptoms already described. Complete obstruction results in bacterial infection of the gallbladder (i.e., acute cholecystitis). Obstruction of the common bile duct (i.e., choledocholithiasis) results in obstructive jaundice, and may lead to reflux of the pancreatic duct (leading to gallstone pancreatitis). Choledocholithiasis may also result in infection of the biliary system (i.e., ascending cholangitis), which has been associated with severe sepsis and high mortality.^2^ Gallstones are also a risk factor for gallbladder cancer, although this is very rare (found in 0.3% of patients with gallstones).^1^

In patients with asymptomatic cholelithiasis, 1-4% will have their conditions progress annually to complications.^1^ Due to the morbidity and mortality associated with the more severe complications of gallstone disease, cholecystectomy is generally recommended for lower-risk patients with symptomatic cholelithiasis. Today, most cholecystectomies are performed using a minimally invasive laparoscopic procedure, with rates of intraoperative conversion to open surgery varying widely from 1 to 15%.^3^ An alternative to surgery generally reserved for higher-risk patients includes percutaneous drainage of the gallbladder.^1^ Additionally, some patients with choledocholithiasis frequently require endoscopic retrograde cholangio-pancreatography (ERCP) with retrieval of stones and possible stenting of the biliary system before cholecystectomy. There are currently no medications proven to treat cholelithiasis. Providers can prescribe ursodeoxycholic acid to prevent stone formation in high-risk populations without affecting symptoms.^1^

One older article estimated that more than 700,000 cholecystectomies were annually performed in the US.^4^ The incidence of gallbladder disease has increased by over 20% during the last 30 years in the US and is now estimated to annually cost approximately 6.2 billion dollars.^5^ With the increased focus on cost containment, improved control of prolonged hospital length of stay (LOS) in cholecystectomy patients is paramount.^5^

One major factor used to control costs in surgical patients is decreasing the time period to surgical intervention when needed. Previous studies have demonstrated that decreased time to surgical intervention results in lower hospital costs and is not associated with worse outcomes.^6–12^ It has also been well documented that earlier operative intervention tends to be superior to delayed intervention, especially in the acute cholecystitis population.^6,10–12^ However, agreed upon criteria for “early” surgical intervention continue to be debated.^12^ The literature reveals the most cited criteria as surgery with 72 hours of presentation.^12^ Experts have also demonstrated that early operative intervention is associated with shorter LOS, reduction in mortality, complications (e.g., bile duct leaks and injuries, wound infections, conversion to open rates and blood loss).^6,12^

In 2014, a research group analyzed the factors associated with increased LOS for acute cholecystitis patients (e.g., worsening disease severity and comorbidities.^13,14^ The cholecystectomy patients in this study with longer LOS accrued increased total surgical costs by up to 64% on the fifth hospital day ($11,087 for day of admission, and $18,196 by the fifth day), although postoperative LOS did not change.^7^ Thus, advocating for “earlier” surgery and preventing prolonged LOS appears to be key for achieving most efficient patient care.

To date, studies addressing LOS in biliary disease have primarily focused on a specific etiology (i.e., most often acute cholecystitis), and not the entire range of biliary disease. As a result, there is still limited information regarding the factors impacting LOS in all cholecystectomy patients. In this study, we were therefore interested in examining the factors that influenced LOS for all cholecystectomy patients at a community hospital with the goal of improving perioperative patient care and decreasing hospital costs. Given that the vast majority of cholecystectomies performed at our institution were laparoscopic (i.e., approximately 6% rate of conversion from laparoscopic to open during the analytic period), the authors made no distinction made between open and laparoscopic cholecystectomy cases.

## METHODS

Saint Joseph Mercy Oakland Hospital is 443-bed facility located in Pontiac, MI. The community has a population of 59,889 people, with an average age of 33.5 years, and an estimated per capita income of $16,087. Racial make-up includes 50.9% black, 25.7% white, 15.8% Hispanic, 4.9% 2 or more races, and 2.9% Asian.

Before data collection, the hospital institutional review board had approved the study. This retrospective review examined data from a two-year time period from January 2013 through December 2014 that included patients admitted from the emergency department and undergoing cholecystectomy during their same hospital stay. We identified adult sample patients using a program called Discern Analytics®, searching for the term “cholecystectomy.” Exclusion criteria included patients less than 18 years of age, undergoing a scheduled cholecystectomy or one due to prior work-up, patients not admitted through the emergency department, cholecystectomy performed as a secondary procedure, and pregnant women.

We performed an additional review of 33 subjects with prolonged LOS, defined in this study as a LOS ≥10 days. Thirty-two of these patients were admitted to a nonsurgical service and one subject was admitted to a surgical service. Based on consensus of independent review by the authors, subjects admitted with non-gallbladder disease or had prolonged LOS for reasons unrelated to gallbladder disease or postoperative complications were excluded. Data were collected concerning patient demographics such as patient age, gender, self-reported race, and comorbidities. Further collected data included type of admitting service, time to surgery, pre and postoperative diagnosis, LOS, postoperative morbidity and mortality, and 30-day readmission rates.

All statistical analyses were conducted using SPSS version 22 software. Univariate analysis examined potential predictive factors suggested in the literature to have an effect on LOS.^13,14^ These factors included type of admitting service, presence of comorbidities, preoperative diagnosis, and length of time to surgical intervention. A series of t-tests were then completed to compare LOS differences in these variables. Since the authors also selected time to surgery as a potential continuous variable predictor of increased LOS, a series of Pearson’s r correlation tests were also conducted. The authors collected additional data regarding discharge location (e.g., home, skilled nursing facility, etc.) to evaluate possible relationships between these rates and LOS.

After we determined that sample patients’ admitting service was a significant predictor of LOS, we conducted further analyses. We used a series of Chi-square tests to compare the LOS of patients admitted to surgical or non-surgical services controlling for presence of comorbidities. We entered these comorbidity analytic terms in an analysis of covariance (ANCOVA) as equally weighted covariates with the dependent LOS variable and additional independent variable type of admitting service. Finally, we completed additional t-tests to examine the significance of differences in time to surgery between surgical vs. non-surgical admitting service patients.

## RESULTS

There were 854 cholecystectomies performed at Saint Joseph Mercy Oakland Hospital during the two-year analytic period. Of these, 542 (63.5%) subjects were excluded based on our selected exclusion criteria. The majority of excluded patients were outpatient and/or elective cholecystectomies. Data from 312 (36.5%) sample patients who met inclusion criteria were analyzed.

The demographic characteristics of sample patients can be seen in Table 1. Upon chart review, the average age of the subjects was 54.5 years of age (±20.3, range 18-92 years). Two hundred and eleven (68%) subjects were female, and one hundred and one (32%) were male. The racial profile of the sample was skewed toward Caucasians since although ~51% of the given community population self-reported being African-American, the profile of this sample was: 72.6% white (n = 230), 13.8% African American (n = 43), 6.7% Hispanic (n = 21), 1.0% Asian (n = 3), and 5.4% unknown or other (n = 17). Patients were about evenly split between admission to a surgery service (48.3%, n = 153) or non-surgical service (51.7%, n = 164).

**Table 1:** Sample Demographics (N = 317 Cholecystectomy Patients)

**Demographics for Study Subjects**	
**Average age**	54.2 (+/-20.3, range 18-92)
	
**Gender**	
Female	214 (68%)
Male	103 (32%)
	
**Ethnicity**	
White	230 (72.6%)
African American	45 (14.2%)
Hispanic	21 (6.6%)
Asian	3 (0.9%)
Unknown	18 (5.7%)
	
**Admitting Service**	
Admitted to surgical service	153 (48.3%)
Admitted to nonsurgical service	164 (51.7%)
	
**Comorbidities**	
Chronic obstructive pulmonary disease	12 (5.0%)
Other lung disease	9 (2.8%)
Diabetes mellitus	57 (18.0%)
Cirrhosis	0 (0%)
Other liver disease	3 (0.9%)
Valvular heart disease	0 (0%)
Arrhythmia	22 (6.9%)
Coronary artery disease	39 (12.3%)
Anticoagulation	11 (3.5%)
Congestive heart failure	20 (6.3%)
Previous abdominal surgery	150 (47.3%)

This was a relatively healthy sample of patients. The incidence of individual comorbidities documented were as follows:

- Chronic obstructive pulmonary disease (COPD) (n = 16, 5.1%),
- Other lung disease (n = 9, 2.9%),
- Some form of diabetes (DM) (n = 56, 17.9%),
- Cirrhosis (n = 0, 0.0%),
- Other liver disease (n = 3, 1.0%),
- Heart valve disease (n = 0, 0.0%),
- Cardiac arrhythmias (n = 21, 6.7%),
- Coronary artery disease (CAD) (n = 38, 12.2%),
- Taking an anticoagulate (n = 11, 3.5%),
- Congestive heart failure (CHF) (n = 19, 6.1%), and
- Previous abdominal surgery (n = 150, 47.3%).

As previously mentioned, intraoperative conversion to an open cholecystectomy was n = 14 (4.5%) in this sample. Postoperative complications were also rare in study patients. The incidence of each study complication was as follows: postoperative bile leak (n = 6, 1.9%), surgical site infection (n = one, 0.3%), pneumonia (n = three, 1.0%), myocardial infarction (n = one, 0.3%), intra-abdominal infection (n = three, 1.0%), death (n = four, 1.3%). There were zero instances of biliary duct injury observed in this cohort. We concluded that the size of this sample subgroup with complications was too small to conduct further analyses.

Post-hospital patient disposition frequencies were as follows: home n = 306 (98.1%), subacute rehabilitation n = three (1.0%) and acute rehabilitation n = two (0.6%). Since most sample patients were discharged home, there was no further statistical evaluation. Similarly, the 30-day readmission rate for sample patients was relatively low, at n = 50 (16.0%). Of these readmissions, 25 (50.0%) were for reasons related to gallbladder disease (i.e., defined as any reason directly related to postoperative complaints or complications).

We conducted a univariate analysis to assess factors that affected LOS. Factors found to be significantly associated with increased LOS included admission to a non-surgical service (5.56 days vs. 3.43 days, p < 0.0005) and increased time to surgery (Pearson r 0.434, p < 0.0005). Non-significant factors included comorbidities and preoperative diagnosis. Due to these findings, the authors performed further analyses concerning evaluating admitting service. (Table 2)

**Table 2:** Analyses of Patients Admitted to Surgical Admitting Service Versus Non-Surgical Service*

**Analysis of Admitting Service**			
	**Surgical**	**Non-Surgical**	**p value**
**Comorbidities**			
Chronic obstructive pulmonary disease	1.3% (n=2)	8.6% (n=14)	0.004
Other lung disease	1.3% (n=2)	4.3% (n=7)	0.175
			
Diabetes mellitus	13.1% (n=20)	22.7% (n=37)	0.029
Cirrhosis	0% (n=0)	0% (n=0)	n/a
Other liver disease	1.3% (n=2)	0.6% (n=1)	0.612
Arrhythmia	3.9% (n=6)	9.8% (n=16)	0.047
Valvular heart disease	0% (n=0)	0% (n=0)	n/a
Coronary artery disease	5.2% (n=8)	19.0% (n=31)	<0.0005
			
Anticoagulation	0.7% (n=1)	6.1% (n=10)	0.011
			
Congestive heart failure	2.0% (n=3)	10.4% (n=17)	0.002
Previous abdominal surgery	39.2% (n=60)	54.9% (n=89)	0.007
			
**Time to surgery**	0.94 days (+/- 0.94)	2.39 days (+/- 2.61)	<0.0005
			
**LOS**	3.5 +/- 1.8 days	5.6 +/- 3.0	<0.0005
			
**Readmission**	8.5% (n=13)	23.5% (n=38)	<0.0005

*please contact the corresponding author regarding any questions relating to the data in this table.

As seen in Table 2, patients admitted to non-surgical services were also significantly “sicker,” meaning they were more likely to have one of each of the following comorbidities: COPD, DM, CAD, on an anticoagulation medication, CHF and previous abdominal surgeries (p < 0.0005). However, none of these individual comorbidities were statistically significant when placed into an ANCOVA model. The only p value that remained significant was the type of admitting service, with patients admitted to the non-surgical services having longer LOS (p < 0.005) when controlling for comorbidities. Patients admitted to a non-surgical service also had increased time to surgical intervention (Mean 2.27 days, ± 2.52) compared to surgical service admission patients (Mean 0.93 days ± 0.94 days; p < 0.0005).

## DISCUSSION

In our study, admission to a non-surgical service and increased length of time before surgical intervention were significantly associated with longer LOS in cholecystectomy patients. Based on prior studies, the estimated total savings for the patients admitted to the surgical service in this study setting may have totaled $4,156 in surgery and $6,390 in extra hospital LOS costs.^6–12^ This could have conceivably resulted in an average total per patient cost savings of $10,546.

The overall findings of this study are similar to other surgical conditions in the literature. For example, two studies have demonstrated that patients admitted to a medical hospitalist service with small bowel obstruction had increased length of stay and increased charges as compared to patients admitted to the surgical service.^15,16^ However, the data for gallbladder disease as a whole remains lacking. One study has shown that patients admitted to a surgical service receiving a cholecystectomy for acute mild gallstone pancreatitis had shorter time to surgery, shorter LOS and lower hospital costs compared to patients admitted to the medical service.^17^

In this community-based setting, patients admitted to non-surgical services were more likely to have documented comorbidities with longer LOS and increased time to surgery when controlling for those comorbidities. Since this was a retrospective review, we could not make a clear distinction between admission time and time of diagnosis, or stratify by the type of varied symptoms that patients may have had at time of presentation. For example, providers may have admitted some patients to the hospital with sepsis of unknown origin with the diagnosis of cholecystitis later made. Such patients may have been admitted to a non-surgical service with increased time to surgical evaluation, increased time to surgery and prolonged LOS. Although we did conduct ANCOVA analyses to attempt to control for certain comorbidities, it would have been ideal if we could have actually matched patients in the surgical and non-surgical admitting services to control for these comorbidities. Further studies of this type are warranted.

One possible means of decreasing LOS and hospital costs in this population is implementation of an acute care surgery (ACS) service.^18–20^ This care delivery model involves creation of a specific surgical hospital service for the evaluation and treatment of emergent non-trauma patients admitted under a variety of surgical emergencies. These types of admitting services have been created in the US and Canada to supplement trauma surgeons’ operative workload and provide more timely care to emergency surgical populations.^18,20^

Several suggested benefits of ACS services include improved scheduling and operating suite predictability for surgeons, improved patient access to surgical intervention and improved post-discharge follow-up.^18^ More efficient workup and management of complex surgical conditions has also been shown, with a cost savings potential for health care systems.^19,20^ When implemented for cholecystectomy patients, authors have initially shown reductions in time to surgical evaluation and to operative room, fewer complications and shorter LOS.^21^ In one study, there was a significant relative cost savings of $3,225.00 measured for cholecystectomy patients under an ACS service (i.e., $13,128.00 under the traditional model and $9,903.00 in ACS).^21^ In another study, an ACS service at a larger institution initiated dedicated operating room time that even further improved time to surgery.^22^

There are several limitations to this study. The study was set at a single institution using a smaller retrospective convenience sample. The generalizability of our results to other organizations may be limited, and we may have had an inadequate level of statistical power to detect meaningful relationships possibly detectable in a larger sample. Additionally, the results of this study may have been subject to unmeasured confounding influences.

## CONCLUSIONS

In conclusion, these results indicate that providers may expect longer LOS for many cholecystectomy patients admitted to a non-surgical service with increased length of time to surgical intervention. These results and the findings of earlier studies suggest that certain factors can be targeted to decrease LOS in this gallbladder disease population. One may argue with this conclusion since our sample patients admitted to non-surgical services were “sicker,” although we still found then to have longer LOS and greater time to surgical intervention when controlling for comorbidities. Ideally, ACS can have helped providers achieve a more rapid evaluation of potential “gallbladder patients” in the emergency department by surgical team providers. Further studies are required to evaluate the actual cost savings derived from these types of hospital admission services to optimize the outcomes in this growing population of patients.

### Conflict of Interest

The authors declare no conflict of interest.
